# A Case of Primary Central Nervous System Lymphoma That Developed in a Patient Receiving Fingolimod Therapy for Multiple Sclerosis

**DOI:** 10.7759/cureus.51108

**Published:** 2023-12-26

**Authors:** Kengo Takanashi, Shinjiro Fukami, Jiro Akimoto, Jun Matsubayashi, Michihiro Kohno

**Affiliations:** 1 Department of Neurosurgery, Tokyo Medical University, Tokyo, JPN; 2 Department of Neurosurgery, Kohsei Chuo General Hospital, Tokyo, JPN; 3 Department of Anatomic Pathology, Tokyo Medical University, Tokyo, JPN

**Keywords:** malignant lymphoma, surgical biopsy, systemic chemotherapy, fingolimod, patients with multiple sclerosis

## Abstract

Fingolimod is an oral medication for the prevention of multiple sclerosis relapse, and its efficacy has been demonstrated in several clinical trials. Fingolimod has various side effects, such as arrhythmia and hepatic dysfunction. In addition, there have been rare reports of the development of lymphoproliferative disorders in patients undergoing fingolimod therapy, including primary central nervous system lymphoma (PCNSL). We diagnosed and treated a multiple sclerosis patient who developed PCNSL while undergoing fingolimod therapy. Fourteen months after starting fingolimod therapy, the patient developed aphasia, and underwent biopsy analysis for a lesion displaying a homogeneous gadolinium-enhanced lesion in the left frontal lobe. The lesion was diagnosed as diffuse large B-cell lymphoma by pathological examination. After the diagnosis, the patient received chemotherapy together with methotrexate combination therapy, and the lesion became smaller and the patient’s symptoms improved. Although several autopsy cases of PCNSL in patients who received fingolimod therapy have been reported, there have been few reports to date of patients diagnosed by biopsy analysis.

## Introduction

Multiple sclerosis (MS) is a demyelinating inflammatory disease of the central nervous system caused by autoimmunization of the myelin protein or oligodendrocytes [1].

For the acute phase of MS, pulse steroid therapy is the main therapy. On the other hand, for the prevention of MS relapse, disease-modifying drugs, including interferon-beta, glatiramer, dimethyl fumarate, natalizumab, and fingolimod are used according to the patient’s needs [2-5].

Fingolimod suppresses MS relapse by inhibiting the autoreactive infiltration of peripheral blood lymphocyte T-cells into the central nervous system [6]. However, fingolimod causes several severe side effects that affect brain tissue, such as progressive multifocal leukoencephalopathy (PML) [7]. Additionally, lymphoproliferative diseases, including primary central nervous system lymphoma (PCNSL), have also been reported, although their direct association with fingolimod remains unclear [8,9]. In Japan, there is only one reported autopsy case of a patient with PCNSL, who died during a clinical trial of fingolimod [8].

When a patient undergoing fingolimod therapy has a new lesion in the brain tissue together with edema, whether it is MS relapse, PML, or PCNSL should be determined. However, in some cases, diagnosis is difficult using the results of neuroimaging, the patient’s clinical course, and laboratory data, and hence, biopsy analysis is required.

## Case presentation

**Figure 1:**
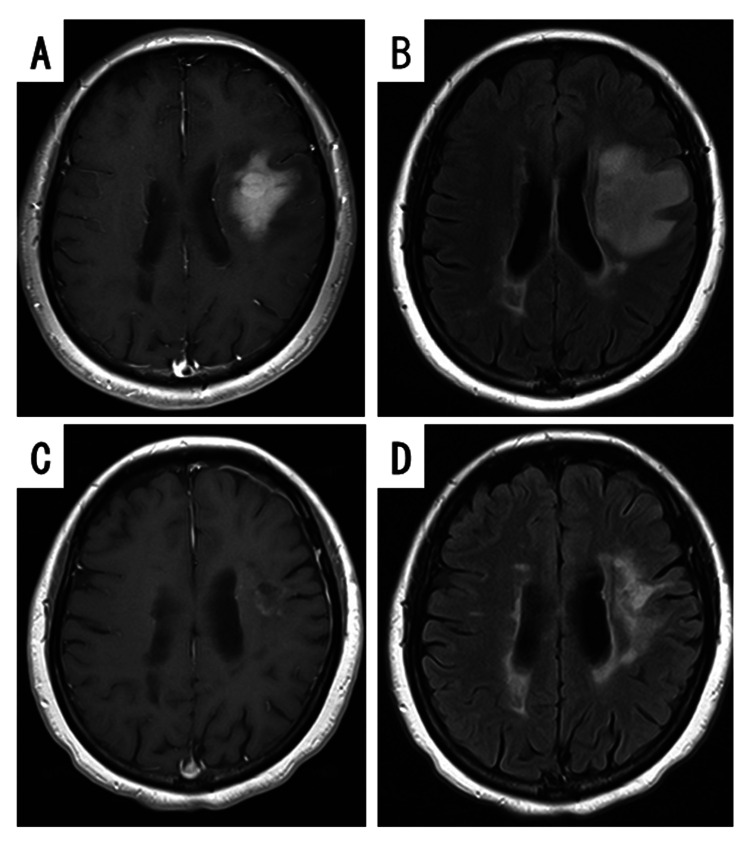
Gadolinium-enhanced (Gd+) and FLAIR MRI before the surgery and after chemotherapy Preoperative MRI displayed a homogeneous Gd+ lesion (A), surrounded by a high-signal area of edematous changes on FLAIR (B) in the left frontal lobe. After the biopsy and three courses of R-MPV therapy, the lesion in the left frontal lobe had almost completely disappeared, although it had a slight contrast (C), and the high-signal area on FLAIR was also significantly reduced (D). FLAIR: fluid-attenuated inversion recovery; R-MPV: rituximab, methotrexate, procarbazine, and vincristine

The patient was a 51-year-old woman with a medical history of hyperlipidemia and MS. She was diagnosed as having MS when she was 27 years old. She presented with glove-sock-type sensory disturbance and underwent steroid pulse therapy using methylprednisolone (1,000 mg/day/x3/cycle). The initial steroid therapy was effective, but the patient continued to experience worsening and remission, and underwent five additional steroid pulse therapies, at 39, 44, 45 (twice), and 51 years of age. She also started taking dimethyl fumarate daily from 48 years of age to prevent recurrence. Subsequently, owing to multiple relapses, her preventive medication was changed from dimethyl fumarate to fingolimod at 50 years of age. After 14 months of fingolimod treatment (0.5 mg/day), she developed motor aphasia, and MS relapse was suspected. Her symptoms were right facial palsy, mild right hemiplegia, and motor aphasia. Brain MRI almost two weeks after aphasia onset displayed a homogeneous gadolinium-enhanced lesion with a maximum length of 37 mm (Figure 1A), surrounded by high signal edematous changes on fluid-attenuated inversion recovery (FLAIR) in the left frontal lobe (Figure 1B).

On diffusion-weighted images, the lesion had a normal signal. Blood tests showed no abnormalities, including Epstein-Barr (EB) virus DNA, and cerebrospinal fluid test results were normal, including the IgG index and myelin basic protein. The differential diagnoses from the MRI results were MS relapse (including tumefactive demyelinating lesions), PCNSL, malignant glioma, and inflammatory PML. An accurate diagnosis was considered necessary to determine the patient’s future treatment plan, and a craniotomy biopsy was performed almost one month after aphasia onset, owing to the need for a large amount of tissue to accurately determine whether the lesion was a neoplasm or caused by a neurodegenerative disease. Frozen section analysis led to a diagnosis of lymphoma, and steroid pulse therapy was started immediately after the operation.

**Figure 2:**
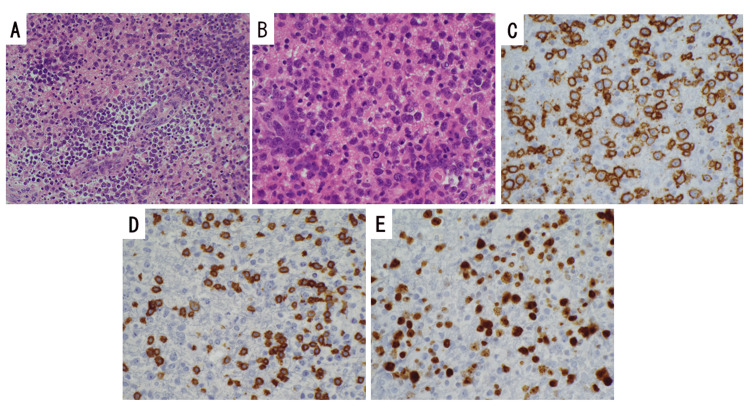
Histopathological data of the brain biopsy diagnosed as PCNSL An H&E stained biopsy sample observed under moderate magnification demonstrated diffuse atypical lymphocytes of medium to large size with irregular nuclei and nucleoli, and atypical lymphocyte aggregation around the blood vessels (A). Upon high magnification, large cells with a high nucleocytoplasmic ratio, and small lymphocyte-like cells with frequent nuclear atypia and mitosis were observed (B). An immunostained biopsy sample observed under high magnification demonstrated that the atypical lymphocytes were CD20-positive (C), indicating DLBCL. A cluster of CD3-positive small atypical lymphocytes was also seen (D). The Ki-67 labeling index was 80% (E). PCNSL: primary central nervous system lymphoma; DLBCL: diffuse large B-cell lymphoma

Microscopically, tumor cells were found to be diffusely invading the neural parenchyma in a perivascular pattern, and infiltration of small lymphoid cells into the neural parenchyma in specific regions was also observed (Figure 2A). Many tumor cells with large nuclei and prominent nucleoli were observed (Figure 2B). Some tumor cells in the lesion also demonstrated mitotic characteristics. Immunohistochemically, the tumor cells showed positivity for CD20 (Figure 2C), and the small lymphoid cells showed positivity for CD3 (Figure 2D). The Ki-67 labeling index was approximately 80% (Figure 2E). The lesion was diagnosed as diffuse large B-cell lymphoma (DLBCL) with mild inflammation. No EB virus-encoding region-positive cells were identified by in situ hybridization. After the definitive diagnosis, fingolimod was discontinued, and three courses of rituximab, methotrexate, procarbazine, and vincristine (R-MPV) therapy, which is a multiple-drug high-dose methotrexate regimen, were administered.

After the three courses of chemotherapy, the lesion had almost disappeared, together with the improvement of neurological symptoms (Figure 1C, 1D). The patient’s symptoms have remained stable, and no enhanced lesions on brain MRI have been detected in the patient, who has not received steroid or fingolimod administration for 19 months after the brain biopsy.

## Discussion

In reports on the adverse events of fingolimod, malignant lymphoma was reported in various organs, such as the bladder, eye, mediastinum, and skin [10-13]. Many of these cases were associated with EB virus infection and were worsened by fingolimod treatment. In a phase 2 study on Japanese patients, among 57 patients undergoing fingolimod treatment, only one patient developed malignant brain lymphoma. This patient was treated with fingolimod for nine months, and died five months after the discontinuation of the drug, following a relapse of severe MS [8]. A subsequent autopsy confirmed that the death was a result of B-cell lymphoma, and a link to fingolimod could not be ruled out. In the present case, as in the case of fingolimod-associated malignant lymphoma described above, the tumor developed after receiving fingolimod. Villano et al. also speculated that the incidence of PCNSL is increased in older patients owing to immunosuppressive and other medications [14]. Although the patient in the present case was young (51 years old), she had been repeatedly treated with high-dose steroid therapy, which may have contributed to her immunosuppressed state. This may also have contributed to the onset of PCNSL. In addition, there was no accompanying EB virus infection or precancerous lesions.

Regarding the pathological features of our present patient, immunostaining detected not only CD20-positive tumor cells but also many CD3-positive small lymphocytes of the T-cell lineage, some of which were polyclonally proliferating, which is unlike typical DLBCL cases and was unique to this case. Although the mechanism of lymphoma development in patients undergoing fingolimod therapy remains unclear, we believe that such an association may exist based on the above information as well as other factors. Recently, Morillos et al. reported a biopsy case of PCNSL after the use of fingolimod, which did not improve after the discontinuation of fingolimod and chemotherapy with methotrexate and cytarabine, and the patient died one year subsequently [9]. To our knowledge, our present case is the first reported case of a patient with fingolimod-associated cerebral lymphoma who was successfully treated with multidrug high-dose methotrexate therapy, both imaging and symptomatically. When abnormal brain lesions are detected in fingolimod users, fingolimod-associated PML should also be considered [15]. Contrast-enhanced lesions on MRI may also be associated with inflammatory PML or PML-immune reconstitution inflammatory syndrome, in addition to MS relapse, which may be difficult to diagnose by imaging alone [16]. This may delay diagnosis and treatment, unlike malignant lymphomas that develop outside of the brain. Prompt diagnosis and treatment, as in our case, can lead to the alleviation of symptoms even in patients with malignant lymphoma. For signal-enhanced brain lesions that are detected in patients during fingolimod use for MS, biopsy analysis should be considered with PCNSL in mind, and multidrug high-dose methotrexate therapy should be initiated as soon as possible.

## Conclusions

The efficacy of fingolimod in MS patients is very high. Contrast-enhanced lesions in the brain are usually suspected to be relapses of MS, but other neurological diseases, such as inflammatory PML and malignant lymphoma should also be suspected, although the direct association between fingolimod use and the development of cerebral lymphoma remains unclear. The early identification of drug-associated malignant lymphoma or PML is important, particularly in patients who are being treated with fingolimod or have a history of taking fingolimod. Early biopsy analysis is very important and useful for lesions such as the one in the present patient because it can determine whether or not the patient should continue taking fingolimod and receive additional chemotherapy.
